# “Everybody’s problem but nobody’s problem”: Qualitative study on integrating smoking cessation and mental health services in Singapore

**DOI:** 10.1371/journal.pone.0322786

**Published:** 2025-05-07

**Authors:** Yee Ling Lok, Grace Ping Ping Tan, Mythily Subramaniam, Yvette van der Eijk

**Affiliations:** 1 Saw Swee Hock School of Public Health, National University of Singapore, Singapore, Singapore; 2 Institute of Mental Health, Singapore, Singapore; Universita Cattolica del Sacro Cuore Sede di Roma, ITALY

## Abstract

**Background:**

Although quitting smoking benefits mental health, people with mental health conditions tend to have higher smoking rates and more severe tobacco use disorders. Integration of smoking cessation into mental healthcare, or vice versa, could help to meet the needs of this population. While Singapore offers specialist smoking cessation and mental health services, it is unclear how these services address the needs of people with comorbid tobacco use and mental health issues. This study aimed to explore the integration of smoking cessation and mental health services in Singapore from the perspective of healthcare professionals.

**Methods:**

We conducted one-on-one semi-structured interviews with 21 Singaporean health professionals with expertise in mental health (n = 5), smoking cessation (n = 5), primary healthcare (n = 3), specialist respiratory or emergency care (n = 3) or health systems and policy (n = 5). We recruited participants from the authors’ professional networks and subsequently via snowballing. We used inductive coding methods to identify themes that emerged from the data.

**Results:**

Health professionals were divided on whether smoking and mental health are sufficiently interconnected to justify more integrated or tailored services. Smoking cessation and mental health were generally approached in a siloed manner, reflecting systemic barriers to integration as well as ranging levels of awareness among health professionals on the association between smoking and mental health. While some participants welcomed the integration of smoking cessation and mental health services as a more convenient, effective and equitable way to address the needs of this population, others deemed it unnecessary and viewed smoking as a lifestyle habit, distinct from other mental health issues.

**Implications:**

There is a need to educate health professionals on smoking as a mental health issue and to consider more tailored programmes designed to address smoking cessation and mental health needs simultaneously.

## Introduction

Tobacco use disorder, recognised in the fifth Diagnostic and Statistical Manual (DSM-5), is often comorbid with other mental health disorders. People with a mental health condition are 2–4 times more likely to use tobacco than those without [1–3], especially those with depression [4], bipolar disorder [5,6], anxiety disorders [7,8], substance use disorders [9], or schizophrenia [4,10–12]. In this paper, we cover a spectrum of severity when discussing mental health conditions: on one end those living in the community with mild or well-managed symptoms, and on the other psychiatric inpatients with severe symptoms.

In Singapore, a 2020 study estimated a smoking prevalence of 39.5% among the psychiatric population, 2.4 times higher than that in the general population [4]. People who smoke and who have a mental health condition tend to have more severe tobacco use disorders and withdrawal symptoms [13–16], greater difficulties in quitting [17–20], and tend to be more reluctant to quit as they commonly view smoking as a self-medication [15,16,21]. In a 2022 systematic review, mental healthcare practitioners perceiving smoking as a social norm or low priority issue for patients with mental health disorders was identified as a barrier to encouraging patients to quit smoking.[22] However, it is unclear whether such views prevail in Singapore mental healthcare settings.

While quit programmes often emphasise the physical benefits of quitting, such as reduced lung cancer risk, quitting also improves symptoms of stress, anxiety, and depression [23–28], and improves overall mood and quality of life [29]. Since smoking affects drug metabolism, quitting can reduce the dosage of psychotropic medications by up to 25%, resulting in fewer side effects [30]. Evidence suggests that people who smoke and who have a mental health condition are as motivated to quit and achieve similar abstinence rates in treatment programmes as those without a mental health condition [4,18–20,31–36]. Thus, smoking cessation programmes targeted to this population could yield positive and more equitable health outcomes. Integrating smoking cessation into mental healthcare, or vice versa, may improve the reach of these services and raise the priority of smoking cessation as part of a broader agenda to improve mental wellbeing in the population [30].

Singapore offers various quit services including the national ‘I Quit’ programme which targets the wider community [37], and more specialised programmes targeting employees and hospital inpatients [38]. Little is known on whether, or how, these services address the needs of people who have comorbid tobacco use disorder and mental health issues. Studies suggest that tailored services, designed to address both needs, are more effective [39–43]. In the US, for instance, integrating smoking cessation services with mental healthcare yielded higher quit rates than smoking cessation alone among those with post-traumatic stress disorder (PTSD). Integrated care was delivered by PTSD clinicians and introduced interventions that addressed PTSD-specific symptoms related to smoking [39].

This study therefore aimed to explore the integration of smoking cessation and mental health services in Singapore from the perspective of healthcare professionals. More specifically, we aimed to explore healthcare professionals’ current beliefs on the relationship between smoking and mental health and how these beliefs may influence the ways in which concomitant smoking and mental health issues are approached in healthcare services, and to explore their views on whether, and how, smoking cessation services and mental health services may be integrated in the Singapore context.

## Methods

### Reflexivity

We, the research team, are four non-smokers from the Saw Swee Hock School of Public Health, National University of Singapore. LYL is a Research Assistant, GTPP is a Research Associate, MS is an Associate Professor also affiliated with the Institute of Mental Health, and YV is an Assistant Professor with a background in tobacco control. We all have experience in conducting qualitative studies in the Singapore context.

### Study design

YV designed the study. To explore the integration of smoking cessation and mental health services in Singapore from the perspective of healthcare professionals, we drew on semi-structured interviews with Singapore-based health professionals, hereby defined as those with expertise in a relevant background. These included mental health services or smoking cessation services, the two specialist services in question, primary healthcare which often acts as a first touchpoint to these services, and health systems and policy due to the more holistic views this can provide on service integration. In addition, we interviewed three experts from specialist respiratory or emergency care based on the recommendations of other interviewees.

### Sampling and recruitment

We used a purposive sampling framework to recruit 21 Singapore-based health professionals with diversity by area of expertise: mental health (n = 5), smoking cessation (n = 5), primary healthcare (n = 3), specialist care (respiratory or emergency; n = 3), and health systems and policy (n = 5). Participants met the inclusion criteria if they were age 21 years or above, had a background in smoking cessation, mental health/wellbeing, primary care service or health services, and agreed for the interview to be audio recorded. We initially identified participants via our professional network. Specifically, colleagues or professional contacts in the field of healthcare provided names of local experts in smoking cessation, mental health, health systems or primary care, and we subsequently invited these individuals for interview. We recruited further participants via snowballing (recommendations from interviewees). GTPP and LYL contacted all participants via email invitations. We reached out to 31 participants, 5 of whom did not reply. Out of the 26 who replied, 21 accepted the interview. We reimbursed each participant with $50 Singapore Dollars in cash. We reached data saturation after 19 interviews, after which we stopped recruiting new participants.

### Data collection

YV and GTPP developed an interview guide (Box 1), with questions designed to explore participants’ beliefs on the relationship between smoking and mental health, how these beliefs may influence how smoking and mental health issues are approached in healthcare services, and their views on whether, and how, smoking cessation services and mental health services may be integrated in the Singapore context. GTPP and LYL conducted all interviews in the English language from August to October 2023 using Zoom conferencing software. Interviews lasted 40–80 minutes each, were audio recorded, and transcribed verbatim using a professional transcription service.

**Semi-structured interview guide.**

**Introduction**

Thank you for agreeing to participate in our study. My name is ____ and I am a researcher in the Saw Swee Hock School of Public Health, National University of Singapore. Our study is looking at the integration of smoking cessation and mental health services in Singapore.

- This interview will take approximately 60 minutes.
- To minimize any links between your identity and the data that will be collected, your name will not be used. You will be assigned a code ___. Please use this code to refer to yourself during the interview.
- Where quotes from the interview are used, we will attribute the quote according to your area of expertise, e.g., ‘Researcher’, ‘General Practitioner’. Please let us know if you have any preference on which descriptor we should use.
- What you share will be kept confidential and only be used for this study.
- The data will be stored securely and only shared with the research team.
- You can choose to withdraw your participation at any time, in which case, the data you provided will not be used and will be discarded.
- To ensure that I do not miss anything that you share, this interview will be audio recorded. Please confirm that you agree to the audio recording.

Do you have any questions before we begin the interview?

Thank you. I will begin the audio recording now. [Begin recording]

For the purpose of the recording, I will repeat my requests: do you agree to be interviewed and audio recorded?

**Questions**

1. Tell me a bit about your professional role and experiences, specifically in relation to smoking cessation or mental health services in Singapore.
2. In your opinion, are there any special considerations for smokers with mental health conditions? Why (not)?
3. How well do you think smoking cessation services in Singapore meet the needs of smokers with mental health conditions? Why?
4. How well do you think mental health services in Singapore meet the needs of smokers? Why?
5. In your opinion, is it necessary to integrate the two services? Why (not)?
6. In your opinion, how might smoking cessation and mental health services be integrated?

**Conclusion**

Is there anything you would like to add before we sum up this interview?

If there are no other questions, I would like to thank you once again for agreeing to participate in our study. Thank you for your time!

### Analysis

GTPP and LYL independently coded the transcripts in NVivo using inductive thematic method, to identify themes that emerge from the data and enable the surfacing of new or unexpected themes [44]. We developed an initial codebook with inductive codes, then independently double-coded all transcripts and compared to ensure consistency. We combined similar codes into new codes and added them to the codebook, with discrepancies reviewed and discussed in an iterative process between LYL, GTPP and YV until consensus was reached. We organized codes into themes, sub-themes and overarching categories (see S1 Table for codebook).

### Ethics

This study was approved by the National University of Singapore Institutional Review Board (NUS-IRB-2023–194). All human participants research was performed in accordance with relevant guidelines and regulations. All participants received an extensive briefing of the study and provided written informed consent before participation.

## Results

Participants discussed [1] their views on smoking and mental health, to explore their current beliefs on the relationship between smoking and mental health and how these beliefs may influence how concomitant smoking and mental health issues are approached in healthcare services. They also discussed [2] the current situation with smoking cessation services and mental health services in Singapore, to provide context to [3] their views on whether, and how, mental health and smoking cessation services may be integrated in the Singapore context.

### Views on smoking and mental health

We observed mixed views on the relationship between smoking and mental health, with some health professionals viewing them as two distinct issues and others as interrelated.

#### Two distinct issues.

Several participants contrasted smoking and mental health as two distinct issues, with smoking described as a habit or lifestyle choice rather than a mental health disorder such as those in DSM-5. In the words of a primary care practitioner:

I mean smoker, whether it’s a disease, very hard to call it a disease. Mental health is a well-recognized DSM-4 disease. – Primary Care Practitioner

A smoking cessation counsellor noted that views are shifting, with tobacco use disorder becoming more recognized as a DSM-5 disorder, but described this shift as “kind of slow.” Participants described both lay and professional awareness as low, with a lack of awareness on the negative influence of smoking on mental wellbeing, and with substance use disorders commonly associated with illegal substance use rather than tobacco use. As described by two health systems experts:

Just talk about very top policy makers and advisors, people equate mental health equal to *siao lang* [crazy person]. That’s how they see it… ‘Addiction’ to many of them is, “Oh, it’s drug addiction. Let police deal with them.” – Health Systems Expert

If somebody has a broken leg, I can visually see that, one, you are elderly, you are 75 years old, and you have a broken leg, but for mental health and smoking, they are both invisible. – Health Systems Expert

Some participants attributed the lack of awareness of the association between smoking and mental health to a lack of international evidence associating smoking with mental health issues such as depression and anxiety, or the observation that smoking is not a priority issue in the Singapore mental healthcare setting. As articulated by one participant:

Is smoking cessation top, top, top priority amongst all the priorities they have? Not really… how many people really die of smoking now? Until you can show the cause and effect of smoking, don’t even talk about whether it’s mental wellbeing issue, mental health, it will not gel. – Health Systems Expert

#### Interrelated issues.

Several interviewees, especially smoking cessation counsellors and mental health practitioners, demonstrated an understanding that smoking and mental health are interlinked in that smoking is a common coping strategy against negative states of mind but, at the same time, may contribute to poorer mental health outcomes:

This closet smoking thing, actually I am quite concerned because they’re also usually associated with other kinds of mental conditions. It’s not healthy if someone has to have two sides to them you see. – Smoking Cessation Counsellor

We find that, those say for example, schizophrenia, they do increase the risk of smoking. They do smoke to cope with auditory hallucinations. I mentioned earlier on, there’s this strong association between smoking and how you actually affect the cytokines that catalyze the drug… Those smokers actually they need more higher dosage of drugs to cope with the symptoms. – Mental Health Practitioner

Another mental health practitioner explained that mental health disorders such as schizophrenia increase the risk of smoking because patients smoke to cope with auditory hallucinations. Given the higher smoking rates in psychiatric populations, some primary care practitioners reported that they routinely asked patients with mental health conditions about their smoking history.

Three mental health practitioners and a smoking cessation counsellor shared that quitting smoking improves mental health through various mechanisms. They described quitting as giving patients a sense of confidence and self-efficacy, improved sleep, and improved social ties which in turn improved their mental wellbeing:

There is evidence that if you cut down just smoking, it can improve mental health, yes. I think that for some of these patients, I do see their mental health improving, but I’m not sure whether it’s a result of them quitting smoking or is it because they are compliant with their medications, they’re coming for follow-ups, they’re getting an active life, they’re working. – Mental Health Practitioner

Some participants were of the view that, if smoking is a self-medication, quitting could deepen underlying issues of which smoking may be a symptom:

…if it’s a coping mechanism thing, then that’s where the partnership with a psychiatrist may be helpful, because if it is a coping mechanism, you are actually taking away one of their coping mechanisms. It might worsen their depression or mental health condition. – Health Systems Expert

Some participants thus reasoned that patients needed to reach a point of mental stability before making a quit smoking attempt and that a more holistic approach was required to address not only their smoking, but also the underlying issues related to their smoking:

If you address the underlying mental health issues, not only does the smoking behaviour get improved, hopefully, but also dealing with the underlying medical illness as well. I know that for instance with patients with COPD, bronchiectasis, and other chronic respiratory diseases, they tend to do worse if they have underlying anxiety or depression. – Specialist Care Practitioner

#### Different needs for smokers with mental health issues.

Participants viewed patients who smoke and also have mental health issues as more complex due to their more severe nicotine dependencies and comorbid issues, such as alcohol dependence, which often take priority over quitting smoking. They noted that such patients tend to require treatment timeframes longer than the usual 12–24 weeks, more social support, such as positive peer influence, and more specialist mental health support to successfully quit smoking:

If it is a mental health problem, there is a chance that I could still stick with the person and work through it with them. If it’s a mental illness, there are people better equipped at handling that than me. – Smoking Cessation Counsellor

You need a different approach because firstly, the mental health condition sometimes may mean that they’re not able to fully grasp the concepts that you’re trying to explain for some of them. Therefore, you really need to be quite strategic in what you put forth and how you counsel them so as to make them understand the need for smoking cessation. – Mental Health Practitioner

Some participants, including three mental health practitioners, felt that quitting smoking may worsen a patient’s mental health if other mental health issues were not sufficiently addressed first:

If they’re smoking to cope, but then they are not equipped with anything else, they might not necessarily feel better after [quitting]. – Mental Health Practitioner

### Current situation with quit services

#### I quit programme.

‘I Quit’ is Singapore’s national quit smoking programme, targeted to the wider community. One participant shared that it has a broad reach with 10,000–15,000 signups per year from self-referral, schools, workplaces and primary, specialist and inpatient healthcare, but low quit rates of around 10%. While I Quit provides education and counselling support after an initial assessment of nicotine dependence, it does not provide nicotine replacement therapy (NRT) or mental health assessment. Some participants noted a disconnect between I Quit and other healthcare services, making it challenging to follow up with patients on their quitting journeys. Others noted that the I Quit subsidies were too strict as they required patients to see a doctor and sign up for at least three counselling sessions, resulting in longer waiting times, multiple referrals and patient dropout.

#### Hospital programmes.

Hospital patients, especially those admitted for tobacco-related diseases, are routinely asked about their smoking status and, if currently smoking, referred to quit services which typically include NRT and counselling. While the quit rates in these services were described as high, the dropout rate was also estimated to be quite high. A hospital smoking cessation counsellor observed that referrals to smoking cessation services had surged in recent years due to ‘HealthierSG’, a health systems reform which includes referrals to quit services as a Key Performance Indicator (KPI) in primary care. Some hospitals had a workflow for pharmacists to refer patients to a psychologist without a doctor referral, to streamline the process for patients requiring mental health support.

#### Primary care.

Participants reported that, in primary care settings, smoking history was not routinely taken as it was not considered a priority unless directly related to the patient’s complaint:

…there’s no reason to ask. When patients don’t come for it [smoking cessation], they come for present complaints. ‘I’m here for back pain,’ for example. Why would you want to bring out smoking? – Primary Care Practitioner

Participants noted a mixed capacity to provide smoking cessation and mental health support in primary care settings. A mental health practitioner felt that polyclinics were well-positioned to provide brief counselling for smoking cessation, and a polyclinic practitioner observed that polyclinic nurses were generally motivated to help patients quit smoking. However, due to the strong focus on physical health over mental health in primary care, this setting was described as unsuitable for addressing more complex mental health needs:

At one stage, in fact, [the polyclinic group] was trying to screen everybody with chronic illness to look out for mental illness. The yield is so bad… The routine way of doing things is still very physical health-focused. They don’t look at your mental aspects of it. – Health Systems Expert

#### Other healthcare settings.

Other quit smoking services are offered by retail pharmacies, private quit smoking counsellors, and the military. Retail pharmacies were described as well-equipped to provide NRT and counselling support, but hampered by the lack of subsidy unless patients are referred from I Quit. Private smoking cessation counselling helps clients using a range of methods and were, by a private counsellor, described as sought by people unsatisfied with the public system. Military quit smoking programmes provide counselling and NRT at no cost, as well as a network of smoking cessation ambassadors to support people in their quit attempts, but the scope of these is restricted to military personnel.

#### Barriers to quit service uptake.

Practitioners across the board felt that an important barrier to quit service uptake was the low motivation to quit among people who smoke:

…we do have services, but it’s how much does the patient actually want to quit smoking? The onus is actually on the patient themselves. – Primary Care Practitioner

Two participants reasoned that, while the motivation to quit may be there, patients may not engage with quit services if they believe that they can quit without any help. Others cited the stigma of going to a smoking cessation clinic or being unable to quit without help, or practical barriers including time constraints. Financial barriers were also mentioned, given the lack of subsidy for treatments such as varenicline or the co-pay required for some subsidized services:

Some of them [patients] even directly mentioned that, “Oh, I don’t want to pay the consult fees.” I think the four sessions with my nurses cost about $50, and most of them already have mentioned about the difficulty in paying. – Primary Care Practitioner

### Current situation with mental health services

#### Specialist mental healthcare.

The public mental healthcare service run by the Institute of Mental Health (IMH) offers smoking cessation support to patients via the addictions service. It integrates services from psychiatry, psychology, addictions and social welfare such that patients can see multiple specialists on the same day, to enable a more seamless experience in addressing multiple mental health-related issues. These patients tend to be complex cases, with comorbid mental health issues and more severe substance use disorders and nicotine withdrawal symptoms that are challenging for non-specialist services, such as primary care, to address.

#### Primary care.

While the focus of primary care is generally on physical rather than psychiatric conditions, some doctors are trained to pick up on signs of underlying psychiatric conditions such as inability to sleep, low appetite and low mood which may indicate depression, anxiety, or other conditions. However, according to one primary care practitioner, most family medicine doctors do not see psychiatric patients on a regular basis unless they have underlying physical conditions such as diabetes. Thus, while participants highlighted the importance of screening for mental health in primary care, they also noted the distinction of physical and mental health in primary care as a potential barrier to effectively picking up on underlying mental health issues. In addition, most polyclinics have mental health clinics run by primary care doctors, sometimes with the help of a psychiatrist, to strengthen primary care and reduce the stigma of seeking help for a mental health condition as this route does not require people to engage specialist mental health services. While the HealthierSG initiative is still nascent, the long-term plan is to use it to promote better mental healthcare at the primary care level.

#### Community services.

A smoking cessation counsellor, mental health practitioner and health systems expert described the community mental health services in Singapore as somewhat limited. While Singapore has support services such as Mindline, public awareness of these was considered to be low and accessing such services is still stigmatized. Services for treating substance use disorders, including tobacco use disorder, tend to fall between the cracks as the community mental health services tend to be run by social service agencies, with focus on mood disorders or severe mental illnesses. Thus, these services were, overall, described as limited in their ability to include smoking cessation support.

### Integrating mental health and quit services

Perspectives were mixed on whether mental healthcare and smoking cessation services should be integrated. Participants across the board identified reasons why integration was not appropriate or practical while some participants, notably those with a background in health systems, primary care or smoking cessation, felt that integration was a good idea.

#### Not appropriate or practical.

Some participants reasoned that integration is not necessary as people who smoke are already sufficiently supported by the quit services, and that more complex cases can be referred to social services. In the words of a mental health practitioner:

For some who are really very motivated and all that, they get some psychoeducation from the pharmacists or the nurse and then they can cope with it already. I think it really depends on the needs of the smoker. – Mental Health Practitioner

Some participants, notably mental health practitioners and smoking cessation counsellors, felt that integration was inappropriate as addressing a patient’s mental health condition should take priority over quitting smoking:

Focus, really, is not on the smoking cessation. It’s really on the quitting the drugs and the alcohol… Whilst a lot of the patients that I see may not eventually quit, quite a sizable portion has actually cut down on the amount of cigarettes that they have smoked. To me, that is also a good outcome. – Mental Health Practitioner

People don’t see the seriousness of smoking because it’s not obvious. Let’s say the person smokes, you got lung cancer, nobody knows what. When intoxicated, people can see. That’s the thing, that people don’t see the seriousness of smokers. – Smoking Cessation Counsellor

Some participants were not convinced that those with mental health conditions should be a priority group for smoking cessation. A health systems expert felt that the value of actively screening people with mental health conditions for their smoking status was unclear, given the unclear association between the two. A mental health practitioner, similarly, expressed some skepticism that such efforts were effective and suggested having monitoring systems to track outcomes. Finally, a primary care practitioner highlighted the low perceived urgency of quitting among those with mental health conditions versus thus with respiratory disease:

Why mental health? The respiratory ones are the ones coughing away, and dying from poor asthma control, and COPD. These are the ones that must quit smoking, not the mental health. I’ll prioritize for someone else. – Primary Care Practitioner

Other participants did not favour integration on the grounds that specialists should stick to their area of expertise and may not be trained to offer all types of care under one roof. As put by a health systems expert:

You cannot expect the I Quit program to deal with schizophrenia. Similarly, I don’t think it would be a wise use of a psychiatrist’s time to get him to do smoking cessation when it can be done by a GP and maybe a pharmacist. We are very short on psychiatrists relative to GPs and pharmacists. I think each person should be managing their own area of strength. – Health Systems Expert

Participants also felt that mental health services are too nascent in Singapore to take on the needs of people with comorbid tobacco use disorder and mental health issues. Smoking cessation was described as an issue that, although it could be addressed in different levels of care, is not fully owned by anyone:

At this point, there is a whole suite of people that can help, but it doesn’t really belong to anybody. – Health Systems Expert

Other participants raised issues related to resources and logistics, as integration would require training, time, and buy-in from key stakeholders in various levels of care. Getting this buy-in was described as potentially challenging given the relative low priority given to smoking cessation versus other pressing health or social issues:

If you had to focus on helping a person quit smoking versus helping the next person who, potentially, has to live on the street, what do you go for? Of course, the latter one. Correct. Can [mental health professionals] do it? Sure. Do they have the bandwidth to do it? No. Not in our system right now. – Smoking Cessation Counsellor

#### A good idea.

Some participants welcomed integration as part of a broader move in the healthcare system to integrated, person-centric care. They perceived this as feasible due to new technological advances in wearable technology, which can track mental wellbeing and smoking habits. Some described the system as already being primed for integration of different services via primary care. Integrated care was described as more convenient and beneficial for patients:

If all the services are good for patients, why not just integrate everything so that it’ll be a one-stop hub for patients? Because I think patients, many of the times, I think if you tell them, “No, I refer you here, there, here, there,” I think most of the time, they will be like, “Forget it. It’s very troublesome.” They will not actually seek any help. – Primary Care Practitioner

All the institutions are working in silo. It will be good if we can integrate the services of mental health and smokers together. – Smoking Cessation Counsellor

Two health systems experts and a smoking cessation counsellor described such integrated care as more person-centric as it would cover the psychological part of their smoking behaviour, thereby offering smokers more holistic support. A mental health practitioner reasoned that integration was necessary from a population health perspective, particularly in reducing health inequalities, as those who continue to smoke following reductions in smoking prevalence will be those who are more resistant to quitting:

You’re going to be left with a lot of smokers with all these mental health conditions, which are not being looked after very well because of the very nature of how difficult it is and how intractable it is because of the levels of dependence. – Mental Health Practitioner

#### Depends on severity and patient readiness.

Two mental health practitioners explained that, since not all people who smoke fit into the same profile, not all of them need to see a mental health professional. They reasoned that, instead, a referral pathway would be helpful for those who need it:

It might be a great indicator to know that if this person is smoking three, four packs a day, then it’s very clear that mental health-wise he’s not great. I think for people who are really suffering with mental health, let’s say these kind of mental health challenges, and then they are coping with it by smoking a lot, then definitely yes for these profile of patients. – Mental Health Practitioner

Other participants noted that, whether integrated or not, the effectiveness of these services would depend on the readiness of the patient to change.

#### Ways to integrate.

Participants suggested that there is room to upskill health practitioners across the board to pick up on mental health issues in people who smoke, or in providing quit support to those with a mental health condition:

…it doesn’t matter whether you’re a mental counselor, career counselors, or whatever counselors, or youth counselor. I think there is opportunity for us to create a module of when they are drilling into understanding what has caused this person to pick up smoking. – Health Systems Expert

I think that it’s always a good thing to upskill professionals, either way is a good kind of upgrade to have to understand more... Whether is it for the smoking cessation professionals to gain more knowledge, and expertise in dealing with some mental health difficulties also or vice versa. – Mental Health Practitioner

Participants felt that smoking cessation counsellors can manage patients with mild mental health issues, to help them with emotional difficulties that may affect their smoking. Participants also suggested closer collaborations between specialists, pharmacists, addiction counsellors and other professionals involved in the care of those with comorbid tobacco use disorder and mental health conditions to exchange knowledge and improve patient care:

One thing which I think would be quite useful is to have a case discussion around different specialists, pharmacists, addiction counsellors, addiction specialists to solve and discuss some of these cases and possibly implant some knowledge into the clinicians, a regular CME [continuous medical educations] about smoking. We know about smoking and medical complications, but how to actually manage the patients who are smoking, what is the particular method that they use in the counseling? All these are very useful. – Mental Health Practitioner

One participant also described how such a system could reduce hierarchies that may act as a barrier to providing more effective patient care:

…if patients, let’s say maybe they’re not comfortable to talk to the nurses or doctors, but they ask the pharmacist about nicotine replacement therapy or some drugs, they help them quit smoking. Whether we can empower a pharmacist in that aspect... I guess we should design a system where there shouldn’t be such hierarchy. – Primary Care Practitioner

A mental health practitioner and primary care practitioner felt that primary care professionals were best placed as the integrators as they are trained to help with a wide range of medical issues:

I can use diabetes as a trigger point to help a patient quit smoking. I can use chronic kidney disease management as a trigger point to help a patient quit smoking. I can even use a patient’s rheumatoid conditions to help him quit smoking. Whereas from a specialist point of view, they’re mostly limited to one approach. – Primary Care Practitioner

Other participants, including health systems experts and smoking cessation counsellors, believed that mental health practitioners were best placed as integrators because of their counselling experience and skillset:

I think that a lot of mental health services, people who are able to do this mental health counseling thing or support, I think they are able to do smoking cessation work as well. For a start, their values are right. You have an intention to help the person come out of a certain deep dark situation they are in, and then you want the best for the person. If you’re talking about counseling, they, definitely, do have some basis in the skillset required to counsel. – Smoking Cessation Counsellor

Some participants also highlighted how an integration of mental health and smoking cessation support could tap on community support, such as that offered by the Ministry of Culture, Community & Youth, volunteer welfare organizations, and Health Promotion Board.

## Discussion

Although tobacco use and mental health disorders are interrelated and frequently comorbid [1–3], health professionals were divided on whether smoking and mental health are interconnected and whether services should address both needs simultaneously. Primary care practitioners viewed smoking as distinct from ‘physical’ health complaints and did not consider it a priority unless the patient presented with tobacco-related diseases. Mental health practitioners considered smoking cessation a low priority compared to other mental health issues. Smoking cessation counsellors focused on treating the tobacco use disorder. This siloed approach reflected ranging levels of awareness on the link between smoking and mental health. Consistent with findings from other countries, the myth that quitting smoking does not benefit, or even worsens mental wellbeing, is a common barrier to providing effective care to those with a tobacco use disorder and comorbid mental health condition [39]. As barriers also manifested in the mindset, investing in more tailored programmes will likely require education of health professionals as well as structural change [45].

International studies suggest that investing in such tailored services yields better outcomes. The UK’s Smoking Cessation Intervention for Severe Mental Ill Health Trial (SCIMITAR+), a quit smoking intervention for people with severe mental health conditions, yielded significantly higher quit rates compared to standard quit support [40]. SCIMITAR+ was also cost-effective despite the higher costs associated with tailored care [46]. An adaptation of SCIMITAR+ in Australia was found to be feasible, well-attended, and clinically effective in improving quit related outcomes [41]. Other tailored quit programmes, developed for those with milder mental health conditions, also yielded promising results. These included the UK’s ‘Stop Smoking in Schools Trial’, which integrated smoking cessation support into youth mental health services [42], and Australia’s ‘VicHealth Tobacco Initiative’, which trains mental health professionals to deliver smoking cessation interventions as part of routine care [43].

Thus Singapore’s healthcare system could benefit from incorporating such models into its unique context and infrastructure, to help overcome the barriers identified in our study. We found that, while smoking cessation services are streamlined into specialist mental healthcare at the Institute of Mental Health, the stigma associated with accessing this service may deter people from seeking help [47]. The I Quit programme, being community-based, is well-placed to tailor quit services with mental health and pharmacological support for those with a mild or stable mental health condition. Access to NRT is especially important for people with mental health conditions, as they tend to have more severe nicotine dependence and withdrawal [13–16] and greater difficulties in quitting [17–20]. We found that the current system for NRT subsidy in I Quit is not streamlined, resulting in patient dropout. Thus, I Quit could yield more equitable outcomes by streamlining its system for providing free NRT from a user perspective.

## Limitations

As recruiting health professionals in Singapore can be challenging, we relied on recruiting via our professional network and recommendations from our interview participants which may have biased our findings. Findings may have also been subject to social desirability bias. Nevertheless, our study provides rich, in-depth insights into the nuances of the issue from the perspective of a range of healthcare professionals.

## Conclusions

Levels of awareness on the relationship between smoking and mental health vary, and smoking cessation and mental healthcare tend to be approached in a siloed manner in Singapore healthcare. These reflect barriers in mindset (recognising tobacco use disorder as a mental health issue) and system (providing integrated or tailored services). Addressing the intersection of tobacco use disorder and mental health in Singapore requires a more integrated approach, with education of healthcare professionals and more tailored programmes designed to address smoking cessation and mental health needs simultaneously.

## Supporting information

S1 Table Codebook with codes organised into themes, sub-themes and overarching categories, sample quotations for each sub-theme, and participants included in each theme. (PDF)

S1 Checklist SRQR Checklist. (DOCX)
