# Supplementary material for: “Everybody’s problem but nobody’s problem”: Qualitative study on integrating smoking cessation and mental health services in Singapore
Source: PLoS One. 2025 May 7;20(5):e0322786. doi: 10.1371/journal.pone.0322786 (PMC12057880; doi:10.1371/journal.pone.0322786)
Supplement: S1 Table — (PDF) [file pone.0322786.s001.pdf]

Table S1: Codebook with codes organised into themes, sub-themes and overarching categories, sample quotations for each sub-theme, and participants included in each theme. Abbreviations: HS=health systems expert, MH=mental health practitioner, PC=primary care practitioner, SC=smoking cessation counsellor, SC=specialist care practitioner.

Theme	Sub-theme	Participants	Sample quotations
**Views on smoking and mental health**			
Two distinct issues	Smoking as a lifestyle choice	HS02, HS04, MH02, PC03, SC04	HS02: [on why approach to smoking is more top down:] …smoking is a choice, whereas mental illness is not a choice, right? It's an illness. You are given an illness…it [smoking] is never seen as an illness for smoking cessation... It is seen as a lifestyle. That's the challenge.<br><br>MH02: A lot of the patients, because this is a long standing habit they see as a habit, not as an addiction.<br><br>PC03: I mean smoker, whether it's a disease, very hard to call it a disease. Mental health is a well-recognized DSM-4 disease.<br><br>SC04: It's only I think in the past five years then there is a shift whereby they start to look into this as a nicotine addiction really under DSM-5 as addiction. Which is kind of slow, this shift.
	Lay awareness is low	HS01, HS02, MH01, MH03, HS03	HS01: It's not that well-known. I think there could be some more messaging or public awareness surrounding this.<br><br>HS02: Just talk about very top policy maker and advisors, people equate mental health equal to *xiao lang* [mentally ill person]. That's how they see it… 'Addiction' to many of them is, "Oh, it's drug addiction. Let police deal with them."<br><br>HS03: I don't think people actively think that smoking is causing these things to be worse because if I have anxiety and I have depression, the two most common mental health conditions, I actually think smoking is a protective factor. There is actually no impetus for me to want to quit smoking because then it creates one more problem in my life.
	Professional awareness is low	HS01, HS02, HS03, MH01, MH03, MH04, SC01	HS02: They know the risk of smoking, but do they see it as something that they must put in so much effort to create a war? They don't. I don't think they see that… Smoking is like a factor that leads to mental illness... They're tackling it from the other way round… we don't approach things from social determinant perspective.<br><br>HS03: If somebody has a broken leg, I can visually see that, one, you are elderly, you are 75 years old, and you have a broken leg, but for mental health and smoking, they are both invisible.
	Lack of evidence	HS04, MH04, PC03, SP01	HS04: …the problem is that literature is not rich enough internationally.<br><br>PC03: I don't know whether there's research to show that patient with depression got higher risk of smoking compared to someone without depression.<br><br>SP01: Most of my patients with chronic disease have some form of anxiety or depression, diagnosed or undiagnosed. Whether there's a link between that and their smoking behavior, I'm not too sure to be honest with you.

	Smoking not a priority issue	HS02, SC01	HS02: Is smoking cessation top, top, top priority amongst all the priorities they have? Not really… how many people really die of smoking now? Until you can show the cause and effect of smoking, don't even talk about whether it's mental wellbeing issue, mental health, it will not gel.<br>SC01: …those who have mental illness tend to focus on dealing with the mental illness. Smoking cessation is not a top priority for them.
Interrelated issues	Smoking & mental health interlinked	MH01, MH02, PC02, SP01, MH05, SC02, HS03, SC04, SC03	MH01: Addictions and mental illness are closely linked. We believe there are factors that contribute to this. Firstly, patient with mental illnesses sometimes maybe smoke to treat the symptoms of the mental illness, the nicotine that I mentioned earlier, it gives them a kick, a high. Sometimes it can help them cope with some of the symptoms of the mental illness like schizophrenia, because some of them feel depressed, they smoke in a way to make them temporarily feel better. We also mention how it's also possible for the addictions to contribute to mental illness.<br>MH02: We find that, those say for example, schizophrenia, they do increase the risk of smoking. They do smoke to cope with auditory hallucinations. I mentioned earlier on, there's this strong association between smoking and how you actually affect the cytokines that catalyze the drug… Those smokers actually they need more higher dosage of drugs to cope with the symptoms.<br>PC02: Patients with mental health conditions, it's not uncommon that many of them also smoke… especially those with anxiety disorders, depression, even schizophrenia… so, in the consults, we would also make a point to ask about their smoking history.<br>MH05: There is evidence that if you cut down just smoking, it can improve mental health, yes. I think that for some of these patients, I do see their mental health improving, but I'm not sure whether it's a result of them quitting smoking or is it because they are compliant with their medications, they're coming for follow-ups, they're getting an active life, they're working.<br>SC04: This closet smoking thing, actually I am quite concerned because there's also usually associated with other kinds of mental conditions. It's not healthy if someone has to have two sides to them you see.
	Quitting benefits mental wellbeing	MH01, MH02, SC02	MH01: Quitting tobacco often leads to improved physical health, which is important for mental health. It's the fact that they can quit smoking, it gives them confidence about what they are going through. Gives them a sense of self-efficacy, and this may be beneficial for them in tackling their mental disorder.<br>MH02: I'm seeing quite a number of patients with insomnia problems. That is where I recognize that, "Hey, actually smoking is a problem." …If they bring it [smoking] forward, they can sleep well. They start to have this trust in you. That is where actually you can tell them, maybe you can try cut down slowly, maybe in the next three months you'll smoke instead of five you smoke four.<br>SC02: The factors could be psychosocial… Then you also find that the mental health also show improvement. Then they also quit smoking or they smoke lesser. Also, it could be the other way. They stop smoking, then the family say, "Hey, actually, I saw improvement. You stopped smoking." Then there's less pressure from the family.
	Smoking is a self-medication	HS02, PC03, MH05, SC02, HS03,	MH05: A lot of them don't really have a mental health condition diagnosed per se, but a lot of them are facing stress with their lives... stress is quite often quoted as a reason, and they feel that smoking actually-- even though it mafy be a logical fallacy, maybe it's just a feeling, but they feel that it helps them cope.

		SC04, SC01, SC03	HS03: You need to know the reasons for their smoking. If it's a habitual thing, if it's a coping mechanism thing, then that's where the partnership with a psychiatrist may be helpful, because if it is a coping mechanism, you are actually taking away one of their coping mechanism. It might worsen their depression or mental health condition. If the person is doing it out of habit, that means the person always smokes after a meal, if you take that away, it doesn't affect his coping mechanism for his depression or anxiety, then that is fine.<br><br>SC04: I would think that from the population I have seen, whether it is a patient of low or high economical class, they usually have an underlying psychosocial need that is not met. Maybe they have a lot of family problems, they have some healthcare conditions that is poorly managed. Usually, it's related to mental conditions. It's like a dual aspect. You cannot just look at the smoking.<br><br>SC01: What I realized, as a broad observation is smoking, it's a manifestation of a lot of things that's happening in their life. It's a lot of stressors, relationship problems. It's like the tip of the iceberg, and you look a little bit deeper and you see very, very deep problems that sometimes we don't even know if we can solve in our role as smoking cessation counselors.
	Mental stability needed to quit	HS05, SC04	HS05: If the patient is stressed, the pharmacist will not recommend the patient set the quit date for the time when he's stressed. The decision of setting a quit date is actually done in partnership with the patient.<br><br>SC04: If the patient is stable, usually they will do very well for their smoking cessation journey, I would say. Then they really can put more effort in… they will be more cooperative and to work with us. If they're not stable at that time point, and normally they don't want to even think about trying to quit, they actually will smoke more.
	Need more holistic approach	HS01, MH01, PC02, SP01, SP02, MH05, SC04, SC01, SC03	HS01: I think when moving towards that direction, I think it's useful to, I mean, there's quite obvious linkages between these two issues, smoking and mental health. I do believe there's moves towards linking these.<br><br>SP01: Because smoking is not just a chemical addiction, but it's also a behavioral addiction as well. I think definitely there's room for play in these respects. Definitely there are some underlying reason why people continue to smoke or pick up the smoking in the first place.<br><br>SP01: If you address the underlying mental health issues, not only do the smoking behavior gets improved, hopefully, but also dealing with the underlying medical illness as well. I know that for instance with patients with COPD, bronchiectasis, other chronic respiratory diseases, they tend to do worse if they have underlying anxiety or depression.<br><br>SP02: When we deal with the mental issues, I think you are dealing with the root cause also because they can go down further to analyze what is the really triggering factor for this mentally for them to have this stress factor. When you treat the stress factor, then you treat the disease at the same time.<br><br>SC04: …if MOH really wanted to cut down the smoking prevalence to… less than 10%, then they have to understand that it is not purely a smoking issue. Why the person is smoking in the first place and why the person is still smoking despite whatever, there is always a root cause. You need to treat the root cause.
Different needs for	More complex	MH05, SC02, MH02	MH05: I think definitely there's a distinction because from what I've seen so far, the smokers with mental health conditions, firstly, they tend to smoke more heavily than the general population…. a lot of them experience a lot of withdrawal symptoms when they try to quit. Can you imagine smoking 100 sticks of cigarettes or bidis a day? The amount of nicotine dependence, it's really quite different.

smokers with MHI			SC02: Some will say "No, no. I think my smoking is not that bad. My alcohol is my primary concern." Then we work with the alcohol first. Some will say, "No, no. I think my alcohol is not that bad. I'll work on my smoking." Some, they're willing to work both. It's not easy, but it's manageable to do both also.<br><br>MH02: Talking about schizophrenia, sometimes they do cope (with) all these disturbing symptoms by smoking. Of course, smoking they will actually reduce the bioavailability of the psychiatric medication that will render it not effective, so they may need a higher dosage, so all these are a strong consideration.
	Need longer treatment	MH01, MH05	MH05: I think that for patients with mental health conditions, unlike the general public who may be able to achieve zero cigarettes in, let's say, a certain number of weeks, maybe 12 to 24 weeks, they are able to do that. I think it's very hard for the mental health folks with, let's say, especially schizophrenia, substance abuse population, to be able to hit that milestone in that same time frame.
	Need more social support	SC03	SC03: If let's say you mix around smokers or alcoholics most of the time, of course, it will be harder for you to quit. That's why sometimes the organization actually arrange them to work at a certain place, and they encourage them to stay together with the same group of people so that they can quit together, so you can have each other to support.
	Need different expertise	MH05, SC02, SC01	MH05: You need a different approach because firstly, the mental health condition sometimes may mean that they're not able to fully grasp the concepts that you're trying to explain for some of them. Therefore, you really need to be quite strategic in what you put forth and how you counsel them so as to make them understand the need for smoking cessation.<br><br>SC02: Some of the symptoms, it could be their way of-- they didn't have the coping mechanism. The psychologist will work with them to do some form of relaxation or how to manage anxiety, look at other resources how to help them.<br><br>SC01: Depending on the severity of the mental health problems, yes, because I tend to make a judgment on whether it's a mental illness or a mental health problem. If it is a mental health problem, there is a chance that I could still stick with the person and work through it with them. If it's a mental illness, there are people better equipped at handling that than me.
	Quitting may worsen mental illness	MH01, MH04, SP02, MH05, HS03, SC03	MH04: If they're smoking to cope, but then they are not equipped with anything else, they might not necessarily feel better after that.<br><br>SC03: It can be quite challenging for us because we fear that… if I stop them from smoking, they have no other way to manage their stress and I'm always at this concern that it may exacerbate the baseline mental health, maybe depression or psychotic.<br><br>MH01: …a patient who is quite severely depressed and also a smoker, usually we will treat the depression with the appropriate treatment like antidepressants, psychotherapy first. Why this is so is because when they are very depressed, they usually have very little motivation to do anything else, including quitting smoking. We will focus on what is more serious, what is more severe first.<br><br>SP02: We will always pay attention on their mental health problem, and let their mental health be settled instead of taking off the smoking and causing them more stress.

			HS03: Let's say you have depression and you are smoking, you probably want to deal with the depression first… You may argue that smoking actually is a form of coping mechanism for the patient who is undergoing depression. By taking away the coping mechanism, it might become even more challenging for the person to recover and rehab from the depression.
**Current situation with quit services**			
I Quit programme	Broad reach but lower quit rate	HS01, HS04, SP01, SC01	HS01: [I Quit targets schools and the general population:] Every year I Quit usually gets about 10,000, 15,000 signups... [quit rate] varies. Hospital programs, inpatient programs tend to have a higher quit rate… For I Quit rates, generally, they've been quite stable around about 10%.<br><br>SP01: HPB has strongly advised us [hospital] to refer all patients to join the I Quit program.
	Focus on education and counselling	HS01, HS04, SC04, SC01	HS04: When they come into the I Quit program and state their willingness to sign up, they will go through a Fagerstrom test to assess their level of addiction to nicotine. Then from there they are being advised by our algorithm to offer them certain pathways. Otherwise, only advice for them, but they can choose otherwise. There are three pathways: SMS, tele-counselling, or face-to-face counselling. We don't fix the pathway because this place is really very lifestyle-based also.
	No mental health screening	HS01	HS01: I think at the moment there's more geared towards how addicted you are to smoking, triggers for smoking, that kind of thing, rather than looking at other aspects, for example, mental health issues.
	Limited follow-up	HS01, SP01, SC01	HS01: Now we're moving towards Healthier SG… I enroll with the GP, then they'll refer me to I Quit, and then that's where you will get the linking of your health data in the system.<br><br>SP01: Right now what we are trying to get is essentially to work together with I Quit to get data back again. Because we also like to know that the patients that we refer, how many actually successfully quit.<br><br>SC01: There is a big limitation on what we can do as a vendor. The ability to stay in touch and all that is not available to us. Especially when you're dealing with youth, there are very strict criteria and restrictions that we are given as external instructors, largely to protect the kids.
	Subsidy	HS01, HS04, HS05, SP01, MH05, SC05	HS01: All the programs [in I Quit] are covered and provided for. For example, within workplaces, there may be some cost sharing, maybe 50-50 cost sharing in place. Usually, these are all taken care of. All the costs for counseling are taken care of.<br><br>SP01: The personal feeling that we've shared with HPB and MOH with regards to their subsidy is always that it may be less help. It actually may contradict the help that they are trying to give, because they got very strict criteria for subsidy to happen. I.e you need to sign up for three counselling sessions and only then some subsidy can kick in and things like that. You must see a doctor. You must go to a government institution…[the organisation] has been trying very hard to rope in the GPs and to rope in the retail pharmacists to help with smoking cessation efforts as well. The retail pharmacies have actually agreed to help out with this already. With this subsidy being so strict, what happens is then the retail pharmacies and the private doctors that are keen to help out won't be able to get the subsidy. Hence then patients will then be referred back to us again, which will then create a longer wait time.

Hospital programmes	Active referrals to quit service	HS01, MH02, MH04, PC03, SP01, SP02, SC02, SC04, SC03	SP01: This current workflow is when we identify smokers, then we will ask them, are you keen to see a smoking cessation counselor? If they agree, then we'll put up the referral.<br><br>SC04: There's a surging referral due to the Healthier SG KPI. I think there are more active referrals from everywhere [inpatient and outpatient].<br><br>SC03: For inpatient, of course, they're usually admitted for some reason. If it's a smoking-related disease, like a stroke or MI then it's actually in their workflow to refer to us if patient is agreeable. Then, the rest is generally for any smoker who are admitted, they will just have a quick screening by the nurses, and then if patient is available, then they'll see them before they go home.
	Subsidy	SC05	SC05: [On whether hospital quit programmes cover NRT:] Hospital, yes.
	High dropout and higher quit rates	MH01, SP01, MH05	MH01: I would estimate that there's quite a high dropout rate.<br><br>SP01: For my own hospital, the quit rates have been really good… we attribute it to the fact that they are hospital-based. Most of our patients had a significant life event. That's why they come to you to quit in the first place.
	Screening smokers for mental health	SP01, MH05, SC02, SC04	SP01: [referring to hospitals:] We do try to pick them [mental health cases] up if we can, then we will make the referral to the psychiatrist and the psychologist.<br><br>SC02: Some of them, they may have some mental health issues. Some could be family-related, social issues, some are stressful factors. Then, they're using smoking as a way of coping... we ask them further, "What do you mean by stress?" First, "What stress are you facing now? From there, you get more history from them. Then, you find that actually this stress, it's not just stress... It could be signs of depression, so you refer to psychiatrists for follow-up.
Primary care	Smoking history not always taken	HS02, PC02, PC03, SP02	HS02: [in polyclinics:] …unless your chronic illness is not being managed well, they will not really pay attention to that.<br><br>PC03: I suppose, there's many reason why [smoking history is not reported], because there's no reason to ask. When patients don't come for it [smoking cessation], they come for present complaints. "I'm here for back pain," for example. Why would you want to bring out smoking? Do you smoke by the way? For no reason. Nothing wrong to ask that, but no direct reasons.<br><br>SP02: …studies have shown just a brief advice, five minutes' advice can have a quit rate of 3% to 5%... all of us are supposed to take smoking history… Then briefly we'll advise them, "Smoking is bad for you." Then subsequently we will tell them that we have these smoking cessation counsellors who can come and counsel you.
	Smoking not necessarily a priority	PC01, PC02, PC03	PC01: I will not say it's a standard SOP. As a healthcare professional, when you know the patient is a smoker, for the benefits of health, so you offer them, "Have you ever considered stopping smoking or not? Because you know smoking is not good for you," and all that. We will do a brief counseling… [for those with smoking-related disease] we will advise them because the risk for smokers is even higher. That portion, maybe can be found in the SOP.<br><br>PC03: Is it highly utilized? Probably not. The same reason. I mean, whether the doctor screen. So there's already so much to do. I mean, you say smoking is important, but alcohol is important, diabetes is important, everything's important, Healthier SG is important.

	Mixed capacity to provide counselling	HS02, MH01, PC02, SP02	HS02: At one stage, in fact, NHGP was trying to screen everybody with chronic illness to look out for mental illness. The yield is so bad… the routine way of doing things is still very physical health-focused. They don't look at your mental aspects of it.<br><br>MH01: When we talk about primary healthcare setting, we do have well equipped polyclinics and they do have counsellors. In some of them, they might have trained smoking cessation counsellors to assist, and they can have the help of the pharmacist, who can-- just a pharmacist alone, they can prescribe the nicotine replacement therapies, and you can also give brief interventions on the spot. Then on the other hand, you may have a GP running his own clinic without the help of any other health professionals.<br><br>PC02: My nurses in my clinic, they are all very motivated to help our patients quit smoking. From a nurse's point of view, my nurses would see them and then would encourage them and look for the side effects of the nicotine replacement therapy, and also encourage them and motivate them. Our nurses are usually very well-trained.
Other healthcare settings	Retail pharmacies	HS04, SC05, SC01	HS04: If the participant-- if the customer don't come through I Quit channel, it's not an I Quit participant, then they have to pay for their own service.<br><br>SC05: Normally the patients, they'll know, "Okay, I want something to increase my focus." For example, "I'm looking for caffeine pill or something for my exam or something." Normally they'll ask like, "Something to help to improve my sleep." We'll recommend supplements or some herbs to release the stress. To us, we are still very product-based, and also the expectation of our customers are very product-based, and we will advise them lifestyle modification.<br><br>SC01: There are a lot of things that community pharmacies can do. It's a bit slower in Singapore because of the system that we have. [quit support is only subsidised via I Quit; medication can only be prescribed by doctor in which case the doctor will do the quit counselling]
	Private counselling	HS01, SC01	HS01: There are private companies that do cessation counseling... there are some TCM... via hypnosis and stuff like that. There are different methods of quitting, Allen Carr method of quitting.<br><br>SC01: Why would they come to us in private practice? It's because they are not getting something from the public system.
	Military quit programmes	HS03, SC05, SC01	HS03: There's no availability of cigarette sales in any of our SAF camps… the smoking cessation tools such as nicotine replacement therapies are available to them at no cost.<br><br>SC05: NRT are subsidized if you are a serving NS man and the doctor decides that you need to use these gums or patches.<br><br>SC01: …they call it SCORE Ambassadors or in short, they're smoking cessation ambassadors and the aim is to have one for every unit… when they come through the program, the aim is that they will be able to practice various principles that we discuss and they have some foundation theory-wise on what smoking is or isn't.
Barriers to quit service uptake	Smokers not motivated to quit	MH03, MH04, PC01, PC02, SP01,	PC01: …we do have services, but it's how much does the patient actually want to quit smoking? The onus is actually on the patient themselves.<br><br>PC02: …you do not need to spend a lot of time with your patients once they are ready [to quit].

		MH05, SC02, SC03	
	Smokers want to quit without help	SP02, SC02	SC02: They think that they're able to handle themselves, they're able to stop or cut down.
	Stigma	MH01, SP01, SC04	SP01: …the taboo that comes along with smoking and going to a smoking cessation clinic.<br>SC04: smokers are really heavily being judged. I know they're discriminated and they know…It's just a lot of discrimination, or a lot of stigma, "Oh, you don't have real power, that's why you cannot quit."
	Time constraint	MH04, PC02, SP01, MH05, SC04	SP01: I think the main problem, especially for the younger smokers, is time off work to come to the clinic to get reviewed.
	Financial cost	MH01, MH04, PC02, PC03, SP01, MH05	MH01: …treatments for smoking cessation are currently not subsidized. In terms of the medications, we talk about Champix, for example. These aren't exactly cheap.<br>PC02: Some of them even directly mentioned that, "Oh, I don't want to pay the consult fees." I think the four sessions with my nurses cost about $50, and most of them already have mentioned about the difficulty in paying.
**Current situation with mental health services**			
Specialist mental healthcare	Screening and referral to quit services	MH01, MH04, MH05, SC02, SC03	MH01: …we do have our special outpatient clinics and we also do have addiction counselors that work and they run clinics as well. We could arrange for, and then we do also arrange for the patients, they come and see the psychiatrist and also the addiction counselor who can help them with their smoking cessation. If they are in the addiction service, then we try our best to arrange them in the same day so that everything can be integrated as much as possible for them. We also combine the inputs of other professionals like the psychologists. Say they have problems with the stress and anxiety, other things that maybe need a psychologist input. We also have social worker, like those who have family problems, financial problems, they deal with abuse at home, for example, social worker will be there. We do have integration of various professionals from different disciplines under the same roof.<br>MH05: We see adolescents. We see people who smoke 100 sticks. We see people who smoke bidis. We see patients with mental health conditions like schizophrenia who smoke quite a bit as well. Some of them really find it-- they will actually tell you that they have a lot of difficulties quitting smoking because they have a lot of withdrawal symptoms. I think it really depends on how they present to the clinician. That makes the clinician feel that they have quite an intractable problem with smoking and therefore refers them to us.

			SC02: For the pharmacy side, they are not trained in these mental health issues. Those with challenging comorbid mental health, they come with a lot of complex psychosocial problems. In this aspect, then also they need medication. Medication for the mental issue, which only the psychiatrist can prescribe.
Primary care	Screening for mental health	MH01, PC01, PC02, PC03, SP01	MH01: Not all of them may be picked up, especially if the patient doesn't volunteer these symptoms. I think it's important because we look at some studies that look into suicide. They say that actually quite a substantial number of those who actually commit suicide, they actually do see a GP in the month before they end the life. It does suggest that actually something could be done here.<br><br>PC02: I guess from a family medicine point of view, most of them do not see us that often because at least in our practice, we do not see psychiatric patients on a regular basis unless they have other underlying chronic conditions like, for example, diabetes… I think it's a little bit inappropriate to ask them about their past psychiatric illness.<br><br>PC03: …some doctors are trained to also pick up red flags. For example, if they come with can't sleep well, or low appetite, for example, then you have to think about, is it probably due to depression? Of course, it can be due to many other things like cancer, and all these things. In the training, you are supposed to pick up, and screen for low mood when they present with something that may suggest depression, anxiety, or any other conditions.<br><br>SP01: …once identified by the counselors, we have already allowed a workflow that the pharmacists can directly refer to the psychologists to allow this to happen. Rather than go to a doctor, then doctor refer to the psychologist, things like that. It's like a straight line to them.
	Healthier SG	HS02, HS05, PC03, SP01, MH05	HS05: If he's a Healthier SG GPs, for every Healthier SG patient that gets enrolled to him, he will get an annual service fee at $70. In order to claim the annual service fee, it's not merely just enrollment, he also needs to submit certain specific forms of specific data about the patient. One of the things that he has to submit, and one of the things he has to do when he sees the patient is called the health plan, he needs to ask the patient whether he's a smoker or a non-smoker… In about the next one to two years, we're working on mental health and how we can actually use Healthier SG to also promote better mental health care, both by GPs and by Singaporeans the next two years.<br><br>SP01: Healthier SG has put smoking cessation as one of its priorities, so that has helped us.
	Polyclinic services	PC02, PC03	PC03: …most cluster polyclinics have a source of primary care, we call it mental health clinics… these are services that are run by primary care doctors, sometimes with the help of a psychiatrist either onsite or not onsite to co-manage, and to also to strengthen primary care… The overall idea is to reduce stigma, because in the past, lot of stigma or patient don't want to go to IMH.
Community services	Low public awareness	SC04	SC04: I know there are things like the mind lines and things like these, but it's just not prominent enough. It's not actively publicized… two years ago, we wanted to increase the awareness of mental health conditions in Singapore. Even things like, example, sleeping disorders, insomnia, it didn't take place.
	Stigma	HS02, MH01, SC04	HS02: While we have hit a point where now more people are talking about that, especially the youth, I think we haven't hit a tipping point where it becomes quite comfortable for people to say, "Yes, I have mental illness and I need to seek help and when I need help I go and see a therapist." It becomes like a social norm.<br><br>SC04: It's not talked about, it's still like a stigma.

	Do not cover addiction	HS02	HS02: The CREST and COMIT are primarily run by social service agencies… because addiction is out of these, so we don't really touch anything on addiction nor smoking cessation… the focus is still very much on mood disorder, anxiety, and then even perhaps severe mental illness.
**Integrating mental health & quit services**			
Not appropriate or practical	Not necessary	MH04, PC03, SC01	MH04: For some who are really very motivated and all that, they get some psychoeducation from the pharmacists or the nurse and then they can cope with it already. I think it really depends on the needs of the smoker.<br>SC01: I think it is sufficient for most because the people who come forward are not undergoing very serious mental illnesses. I think it's good enough. There are plenty of times when the success of or the ability to help them reduce their stress levels it's not within our field of expertise and not within our ability to handle because those are grant-based social service kinds of things, that even if we wanted to, we can't help. You are reliant on their social worker to work something out for them.
	MHI takes priority over quitting	SP03, MH02, MH03, MH04, PC02, PC03, MH05, SC02, HS03, SC04, SC03	SP03: For this group, smoking doesn't kill them that fast. The alcohol will kill them. Alcohol is also causing a lot of so social issues for them.<br>MH04: Smoking might not actually be the thing to be targeted. It can be one of the things if the patient wants to work on it, but usually we will be trying to help the patient with what's really causing them distress.<br>MH05: Focus, really, is not on the smoking cessation. It's really on the quitting the drugs and the alcohol… Whilst a lot of the patients that I see may not eventually quit, quite a sizable portion has actually cut down on the amount of cigarettes that they have smoked. To me, that is also a good outcome.<br>SC02: People don't see the seriousness of smoking because it's not obvious. Let's say the person smokes, you got lung cancer, this, nobody knows what. When intoxicated, people can see. That's the thing, that people don't see the seriousness of smokers.<br>HS03: …people with mental health conditions, usually smoking is not at the top of their mind, so you need a more specialized, customized approach that takes into account their constraints or their unique considerations. Whereas the smoking services that we have tend to be a bit more general. It's one-size-fits-all in a certain way.<br>SC03: A lot of social support is needed for this patient. They can't quit smoking by just seeing us for once, but back there, they are still suffering with their own illness. I think they need a lot of social support after that, like a job, friends, a group of friends, like a family, to get them back to the right track of their life. If they can't even get a proper job, there's no motivation for them to continue.
	MHI not priority group for quitting	HS04, PC03, MH05, HS03	PC03: Why mental health? The respiratory one are the one coughing away, and dying from poor asthma control, and COPD. These are one that must quit smoking, not the mental health. I'll prioritize for someone else.<br>MH05: Outcomes should be tracked stringently. There should be a system of monitoring… whether such efforts are effective. Again, we need to develop some best practice to guide this kind of service, which I'm not sure where in the world we have the best practice for mental health patients, for smokers.

			HS03: If you don't have the current numbers, then people will say, "Why do I need to actively screen? If I see a person with mental health conditions or I see a smoker, there's no reason for me to actively screen unless you can show me that there is value, or there's such a high association that I should be actively screening. Otherwise, it will not be good standard of care."
	Specialists should stick to their area	HS05, MH04, SP02, SC02, SC01	HS05: You cannot expect the I Quit program to deal with the schizophrenia. Similarly, you cannot expect a psychiatrist-- I don't think it would be a wise use of a psychiatrist's time to get him to do smoking cessation when it can be done by a GP and maybe a pharmacist. We are very short on psychiatrists relative to GPs and pharmacists. I think each person should be managing their own area of strength.<br><br>MH04: I wouldn't duplicate what the counselor does…. the roles are specific so the counselor will primarily work on the addictive behaviors. Let's say if is a person who drinks then to go through the relapse prevention, who work through cutting down towards abstinence, that kind of thing, whereas, my role would be more to work with the comorbid conditions.<br><br>SP02: I am very cautious when a mental health patient comes to me and they're smokers. My emphasis will not be on smoking cessation straight on because I know I cannot use any other means of medications other than just counseling him and asking him in regards to what he thinks about quitting smoking. I will not push too much.<br><br>SC02: Oh, okay. I think those referred to us usually are those comorbid mental health issues. For the pharmacy side, they are not trained in these mental health issues. Those with challenging comorbid mental health, they come with a lot of complex psychosocial problems.
	Mental health services still nascent	HS04, MH02, PC02, MH05, SC02, HS03	PC02: For mental health services, whether is it enough to meet the needs of the smokers, I would say more work need to be done.<br><br>HS03: At this point, there is a whole suite of people that can help, but it doesn't really belong to anybody.
	Resources and logistics	SP03, MH02, PC02, PC03, MH05, SC01	SP03 [drawing parallels with Yishun Health's alcohol programme:] …it's not the lack of funding, it's actually getting buy-in from the ground. We need to be there, we need key stakeholders in the various hospitals to buy in to the program, to feel that the program can do something for their own patients.<br><br>PC03: Training and time. I think time is also big factor. Do you even have time to do all this counseling?<br><br>SC01: If you had to focus on helping a person quit smoking versus helping the next person who, potentially, has to live on the street, what do you go for? Of course, the latter one. Correct. Can they [mental health counsellors] do it? Sure. Do they have the bandwidth to do it? No. Not in our system right now.
A good idea	More integrated care	HS01, HS05, PC01, PC02, SC02, HS03, SC03	HS01: You've got trackers, your wearable technology. Your wearable technology now is able to track markers for depression, mental health, obviously, those physical activity, if you're a known smoker, then it could add that into the mix, it's quite useful.<br><br>HS05: The GP is the natural integrator, and that's why I'm saying we're shifting the care to primary care in healthcare. If it's too complicated for a GP, it's a very severe depression, along with very strong nicotine dependency and other forms of addiction besides smoking, also

			alcohol and it's also illicit drugs or whatever. It's a diazepine and what have you-- Then my answer is of course it'll be better managed in IMH which is already integrating management of mental health with various forms of addiction including nicotine addiction.<br><br>PC01: If all the services are good for patients, why not just integrate everything so that it'll be a one-stop hub for patients? Because I think patients, many of the times, I think if you tell them, "No, I refer you here, there, here, there," I think most of the time, they will be like, "Forget it. It's very troublesome." They will not actually seek any help.<br><br>SC02: All the institutions are working in silo. It will be good if we can integrate the services of mental health and smokers together. Integrate in the service that there should be more maybe collaboration or some exchange.<br><br>HS03: For smoking cessation... It's everybody's business, but it's also nobody's business.<br><br>SC03: I guess we are moving towards that. We are also trying to engage psychologists into our counseling, especially those with what you say, the mental health issue. We will discuss with them how to manage.
	More person-centric care	HS01, HS02, SC03	HS01: I've seen the development of tobacco control for the last 10 years or so from it being where we just used to scare people into trying to quit, I mean, and now it's more about enabling, creating the right environment, supporting them through their quit journey… I think that's the next journey that we're trying to be more holistic approach into smoking as looking at the sources of these triggers as well as giving the actual nicotine addiction itself. I think it's a positive move to integrate mental health issues.<br><br>SC03: At least it can cover the psychological part of the smoking behavior, which is a huge part.
	Necessary for population health	MH05	MH05: [as smoking rates drop:] …you're going to be left with a lot of smokers with all these mental health conditions, which are not being looked after very well because of the very nature of how difficult it is and how intractable it is because of the levels of dependence.<br>If they are really trying to reduce health inequalities, then I would say it's very important, but because it's so difficult to do it and so resource-intensive, I don't know how ready people are willing to do it.
Depends	On severity of case	MH03, MH04	MH03: It might be a great indicator to know that if this person is smoking three, four packs a day, then it's very clear that mental health-wise he's not great. I think for people who are really suffering with mental health, let's say these kind of mental health challenges, and then they are coping with it by smoking a lot, then definitely yes for these profile of patients.<br><br>MH04: I don't think all smokers, not every one of them would fit into a profile that would need specialized mental health help. I don't think you always need to be involving mental health professionals, but it can be helpful if there is a referral pathway, if there is a pathway to get smokers to be seen by mental health professionals if they need to.
	On patient readiness	HS02, HS05, PC03, SC04	HS02: It's very difficult to go out to look for smokers. If they're not ready, it's very difficult to talk to them, and ask them to change.<br><br>SC04: If they're not ready, then you just have to wait for the patient really.
Ways to integrate	Upskill across the board	HS02, HS05, MH02, MH03,	HS02: …it doesn't matter whether you're a mental counselor, career counselors, or whatever counselors, or youth counselor. I think there is opportunity for us to create a module of when they are drilling into understanding what have caused this person to pick up smoking.

		MH04, PC02, SP02, SC05	MH03: I guess then it's whether the mental health professionals are picking up the level of smoking. Whether they are able to differentiate. Actually, not just mental health professionals, even all your heart doctors and your lung doctors, if they are able to pick out that this person is smoking three packs a day, then maybe that would be very indicative of a mental health issue.<br><br>MH04: I think that it's always a good thing to upskill professionals, either way is a good kind of upgrade to have to understand more... Whether is it for the smoking cessation professionals to gain more knowledge, and expertise in dealing with some mental health difficulties also or vice versa.<br><br>PC02: more into the professional aspect of the physicians, whether they are skilled enough to make mental health and even smoking as part of their history taking. To me, at least I practiced family medicine for quite a while. This is the skills that need to be further developed.<br><br>SP02: Definitely, there is room for improvement. Definitely, we have to learn from others also. I will not say, oh, I have a good training, I'm satisfied with whatever training. I think training depends on who taught you the time and next time who is teaching you. When people advance in trainings and in years of experience, then they will have different ways of counseling difficult patients. We hear from different viewpoints of how different people counsel.<br><br>SC05: I think it is good to get all our pharmacists trained to identify tell-tale signs when we need to refer the patients to get proper mental health support. It's like first aider… it's good to get ourselves as a healthcare professional educated, but we may not actually use it. It's just that to increase the awareness.
	Quit counsellors can take mild cases	HS05, MH04, SP02, SC04, SC03	MH04: …they are capable and proficient to provide support. Definitely dealing and helping the patients with emotional difficulties and all that, those are things that they're equipped with as well.<br><br>SP02: I will firstly assess my patient first. I will see currently where are they at their mental health issues. If they are a stable mental health person, I don't mind taking them. With those who are aggressive, very abusive, then I would just leave it as it is.
	Work more closely with specialists	HS02, MH02, PC02, SP01, SP02, HS03, SC04, SC01	MH02: One thing which I think would be quite useful is to have a case discussion around different specialists, pharmacists, addiction counsellors, addiction specialists to solve and discuss some of these cases and possibly implant some knowledge into the clinicians, a regular CME, continuous medical educations about smoking. We know about smoking and medical complications, but how to actually manage the patients who are smoking, what is the particular method that they use in the counseling? All these are very useful.<br><br>PC02: …if patients, let's say maybe they're not comfortable to talk to the nurses or doctors, but they ask the pharmacist about nicotine replacement therapy or some drugs, they help them quit smoking. Whether we can empower a pharmacist in that aspect... I guess we should design a system where there shouldn't be such hierarchy… It can even be initiated by the pharmacists.<br><br>SP01: Having multidisciplinary discussions where we sit down and discuss cases, I think will be helpful also. What the work group has done is also to create a community of practice… we get people involved in smoking cessation work to gather every once in a while, and discuss difficult cases, stories, book clubs, even journal sharing as well. Together we can discuss and then exchange ideas and share opinions and tips. I think by that integration will be better and more seamless and I've seen that being done.

			HS03: In a typical medical setting, let's say you are a person with multiple medical conditions, let's say you have stroke, you have depression, and you have need of a knee replacement surgery, these style cases usually you get a bit of a multidisciplinary team approach where several doctors manage you… we will call these people complex patients and they may benefit from a multidisciplinary approach.<br><br>SC01: …have more social workers, have more psychologists and have them be able to follow up with these people over a much longer time at a fee that is affordable to either the person they're seeing or the organization or government providing this service.
	GPs best placed	MH01, PC02	MH01: …primary healthcare, they play a very meaningful role because the GP will then say, look, you have your medical issues A, B, C, you also suffer from maybe anxiety, depression, and smoking, and I'm here to help everything for you.<br><br>PC02: For my practice, I can use diabetes as a trigger point to help a patient quit smoking. I can use chronic kidney disease management as a trigger point to help a patient quit smoking. I can even use a patient's rheumatoid conditions to help him quit smoking. Whereas from a specialist point of view, they're mostly limited to one approach.
	Mental health workers best placed	HS02, HS04, HS05, HS03, SC01, SC03	HS04: I think the most important is not about smoking cessation training, but more on their counseling background and experience plus also personality. Then from there, the cessation knowledge or any other knowledge can be applied.<br><br>SC01: I think that a lot of mental health services, people who are able to do this mental health counseling thing or support, I think they are able to do smoking cessation work as well. For a start, their values are right. You have an intention to help the person come out of a certain deep dark situation they are in, and then you want the best for the person. If you're talking about counseling, they, definitely, do have some basis in the skillset required to counsel.
	Community support	HS02, MH02, SC02, SC03	HS02: In fact, if you ask me personally, I always felt MCCY does a very good job in community development and this ground engagement, but their ground engagement is with one thing in mind, community development. It's how do we leverage on that, and then infuse in there health-related, smoking-related, and use that as a way to create the dialogue, the awareness?<br><br>SC02: [On volunteer welfare organizations:] A support group will be with very powerful. Gather people face-to-face, not on the phone… So they can learn then from one another.<br><br>SC03: Something like the Brahm Centre without the costs. Then I think the HPB is doing quite well with the activities as well. They have these weekly classes for people to join. Those are good. It's just that we have to encourage patients to join them.
